# A Critical Candidate Node-Based Attack Model of Network Controllability

**DOI:** 10.3390/e26070580

**Published:** 2024-07-08

**Authors:** Wenli Huang, Liang Chen, Junli Li

**Affiliations:** 1 School of Computer Science, Sichuan Normal University, Chengdu 610101, China; wenli.hhuang@hotmail.com (W.H.); chen.liang.cl@outlook.com (L.C.); 2 Visual Computing and Virtual Reality Key Laboratory of Sichuan, Sichuan Normal University, Chengdu 610068, China

**Keywords:** complex network, controllability, controllability robustness, critical candidate nodes, attack model

## Abstract

The controllability of complex networks is a core issue in network research. Assessing the controllability robustness of networks under destructive attacks holds significant practical importance. This paper studies the controllability of networks from the perspective of malicious attacks. A novel attack model is proposed to evaluate and challenge network controllability. This method disrupts network controllability with high precision by identifying and targeting critical candidate nodes. The model is compared with traditional attack methods, including degree-based, betweenness-based, closeness-based, pagerank-based, and hierarchical attacks. Results show that the model outperforms these methods in both disruption effectiveness and computational efficiency. Extensive experiments on both synthetic and real-world networks validate the superior performance of this approach. This study provides valuable insights for identifying key nodes crucial for maintaining network controllability. It also offers a solid framework for enhancing network resilience against malicious attacks.

## 1. Introduction

In the past two decades, complex networks have gained wide popularity and rapid development, forming an independent discipline that encompasses multiple fields, such as network science, systems engineering, statistical physics, applied mathematics, and social sciences [1,2]. Complex network modeling is an effective method for studying complex real-world network systems. This approach not only allows for the analysis of the complex structures of these network systems but also enables the exploration of how to control and utilize them [3].

In the process of pursuing control over complex systems, controllability has gradually become a research hot spot in recent years. The controllability of complex networks is a fundamental issue in complex network systems. Controllability refers to the ability of a network system to be driven from any initial state to any desired target state within a finite time frame, given an acceptable control input [4,5]. Currently, numerous researchers are focused on network controllability, including the study of the controllability of first-order linear and nonlinear systems, traction control, adaptive control, synchronization control, and delayed synchronization in network systems [6,7,8,9,10]. These methods have been applied in research on network controllability.

The controllability robustness [11] of a network refers to the network’s ability to maintain its controllability under random failures and malicious attacks. Both theoretical and empirical evidence suggest that many complex natural and engineered systems are fragile. In the real world, network systems are increasingly and inevitably subjected to various disruptions. Malicious attacks on nodes or changes in network topology can significantly reduce network controllability. For example, in power grids, connection failures or attacks between substations can severely impact network performance. Similarly, in transportation networks, attacks targeting nodes with high betweenness centrality can disrupt normal operations [12]. To enhance network robustness or design more robust networks, it is crucial to analyze and identify key components affecting controllability through node- and edge-removal attacks [12,13,14,15].

Attacks on networks can be categorized into random attacks and intentional attacks. Node-removal attacks are divided into random attacks and malicious attacks. Random attacks involve the random selection of targets, while malicious attacks select the most critical nodes based on network characteristics (such as degree, betweenness centrality, regional centrality [16], k-core [17], and structural holes [18]). These analyses can inform strategies to strengthen networks against malicious attacks or random failures. Therefore, studying the changes in controllability robustness of network systems under attack is crucial for improving system security and enhancing network stability.

Research has shown that among various attack strategies, those based on network characteristics are generally more effective than those based on random attacks. Recent studies have explored and designed many effective attack algorithms. For example, methods for assessing network centrality emphasize the importance of protecting critical nodes to enhance robustness [19]. Node-based attack strategies typically cause more damage to network controllability than edge-based strategies. Nodes are classified into multiple categories based on their impact on control [20]. Studies also indicate that certain topological structures, such as multi-chain and multi-ring configurations, can improve robustness [21]. Furthermore, removing cut vertices can cause more damage than standard malicious attacks, especially in networks with a high average degree [22]. The impact of node classification and failure on network controllability was studied in [23]; the nodes in the network were classified into nine types based on the directions of edges and matching relationships, and the impact of different types of node failures on network controllability was analyzed. Therefore, identifying target nodes for each attack to maximize the disruption of network controllability is a core challenge.

Based on this, an efficient node-removal attack model is proposed in this paper to study the controllability of directed networks. This model aims to minimize network controllability through strategic node removal. The concept of critical candidate nodes is introduced, and their impact on network controllability is examined by developing an attack model based on critical candidate nodes (CCA). Comparative experiments conducted on synthetic and real-world networks validate the effectiveness and efficiency of the proposed model, demonstrating that the CCA model achieves state-of-the-art performance in terms of attack effectiveness and time consumption.

The rest of this paper is organized as follows. Section 2 introduces the preliminary concepts of controllability and controllability robustness. Section 3 presents a detailed illustration of the proposed attack model, CCA. Section 4 demonstrates the experimental results on synthetic and real-world networks. Section 5 concludes this paper.

## 2. Network Controllability and Controllability Robustness

A complex system can be modeled as a directed complex network, which consists of many nodes and edges between pairs of nodes, where a node represents a functional component of the system and an edge represents a link between components. The network can be represented by G=(V,E), where *V* denotes the set of nodes and *E* denotes the set of edges.

For a linear, time-invariant (LTI) networked system ($\dot{x}=Ax+Bu$, where *A* and *B* are constant matrices of compatible dimensions, x is the state vector, and u is the control input), $\left[B\;AB\;A^{2}B\;\cdots \;A^{N-1}B\right]$ is the controllability matrix of the LTI system, where *N* is the dimension of *A* and also the network size. A *state-controllable* system must have a full row rank of its controllability matrix. The concept of structural controllability is a slight generalization of state controllability to deal with two parameterized matrices (*A* and *B*) in which the parameters characterize the structure of the underlying system in the sense that if specific parameter values are ensuring the system to be state controllability then the system is structural controllability. If there is no control input (u) or B≡0, the networked system is uncontrollable; for a network of one-dimensional (scalar) nodes, the control of some nodes ensures the controllability of the network. Network controllability is measured by the minimum number of nodes with control inputs, called drive nodes (DNs). When the network is put into the above LTI system formulation, all the drive nodes can be described by the matrix (*B*).

Network controllability can be measured by the ratio of the minimum number of drive nodes to the total number of nodes as follows:

(1) $$n_{D}=\frac{N_{D}}{N}$$

where $N_{D}$ is the minimum number of drive nodes needed to retain network controllability, *N* is the total number of nodes, and a lower $n_{D}$ denotes the network has better controllability. The maximum matching of a directed network can determine the driver nodes and their minimum number, which quantifies the structural controllability of the network. Specifically, when finding the maximum matching, $N_{D}$ is determined by the number of mismatched nodes, and $N_{D}$ can be calculated in two ways; for structural controllability, it is expressed as follows:

(2) $$N_{D}=\max\left\{1,N-\left|E^{*}\right|\right\},$$

and for exact (state) controllability, $N_{D}$ is calculated as follows:

(3) $$N_{D}=\max\left\{1,N-rank\left(A\right)\right\}$$

where $\left|E^{*}\right|$ is the number of nodes in the maximum matching ($E^{*}$).

The controllability robustness of a network can be evaluated by recording the current controllability under a series of node-removal or edge-removal attacks; the recorded controllability sequence can be regarded as a *controllability curve*, which reflects how robust a networked system is against destructive attacks. The robustness curve under node-removal attacks is calculated as follows:

(4) $$n_{D}\left(i\right)=\frac{N_{D}\left(i\right)}{N-i},i=0,1,\ldots ,N-1,$$

where *i* indicates the number of rounds of attack, $N_{D}\left(i\right)$ is the number of driver nodes needed to retain the current network controllability, and *N* is the total number of nodes in the original unattacked network. To measure the overall controllability robustness of a network, the controllability curves are averaged as follows:

(5) $$R=\frac{1}{N-1}\sum_{i=1}^{N-1}n_{D}\left(i\right),$$

where a lower *R* represents better overall controllability against node-removal attacks for a network.

## 3. Critical Candidate Node-Based Attack Model

The control of the entire network can be achieved with the minimum drive node set (MDS), and the number of elements in the MDS is also the minimum number of driver nodes; there may be multiple equivalent MDSs for a network derived from different control configurations. The possibility of a node ($V_{i}$) being a driver node can be quantified based on the node control capacity, denoted by $\varphi (V_{i})$ [24], i.e., how often the node appears in the MDS. According to the node control capacity, nodes can be classified into three categories, namely critical nodes, intermittent nodes, and redundant nodes. Their definitions are as follows:

(1) Critical node: $\varphi (V_{i})=1$, meaning the node is a driver node in all MDSs. These nodes are essential for maintaining network controllability, and their removal significantly impairs control.
(2) Intermittent node: $0<\varphi (V_{i})<1$, indicating that the node acts as a driver node in some but not all control configurations. These nodes have a variable role in control and can be crucial under certain conditions.
(3) Redundant node: $\varphi (V_{i})=0$, showing the node is not a driver node in any MDSs. These nodes do not significantly impact the network’s overall control capacity, and their removal does not affect the network’s ability to maintain control.

**Figure 1:**
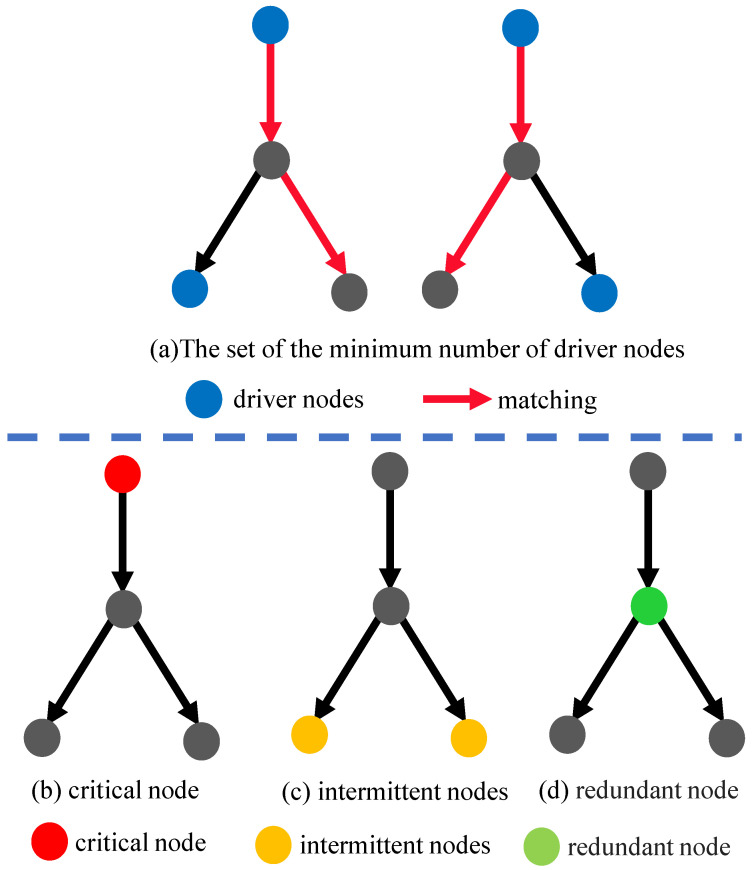
The figure distinguishes essential driver nodes and their relationships (**upper part**) and details different node types’ roles and importance in network controllability (**lower part**). The gray nodes in the Figure 1 are ordinary nodes in the network.

All possible MDSs are illustrated in Figure 1a, with blue nodes corresponding to driver nodes and red arrows indicating the matching relationships. These matching relationships denote the control connections that enable driver nodes to influence other nodes in the network. Figure 1b–d show the three types of nodes in the network.

According to Equation (1), to minimize the controllability of a network, the proportion of driver nodes in the network needs to be increased, corresponding to an increase in the size of the MDS as much as possible; a larger MDS means the worse network controllability. An MDS consists of two parts, namely critical nodes and intermittent nodes, so network controllability can be disrupted in the following two ways:

(1) Attacking the nodes in the network makes the intermittent nodes become critical nodes.
(2) Attacking the nodes in the network makes the redundant nodes become intermittent or critical nodes in the MDS.

The roles of critical nodes and redundant nodes for the control of a network were investigated in [24,25], and it was shown that a sufficient condition for a node to be a critical node is that the in-degree of the node be 0. Therefore, whether a node is a critical node or not is closely related to the in-degree. If the predecessor node of some nodes in the network is removed, then the in-degree of these nodes can be changed to 0, thus making them become critical nodes. In other words, the in-degree of the successor node can be used as an important reference for node-removal attacks.

### 3.1. First-Order Critical Candidate Nodes

#### 3.1.1. First-Order Critical Candidate Nodes (FCCNs)

If a node in the network has a successor node with an in-degree equal to 1, the node is called a first-order critical candidate node.

**Figure 2:**
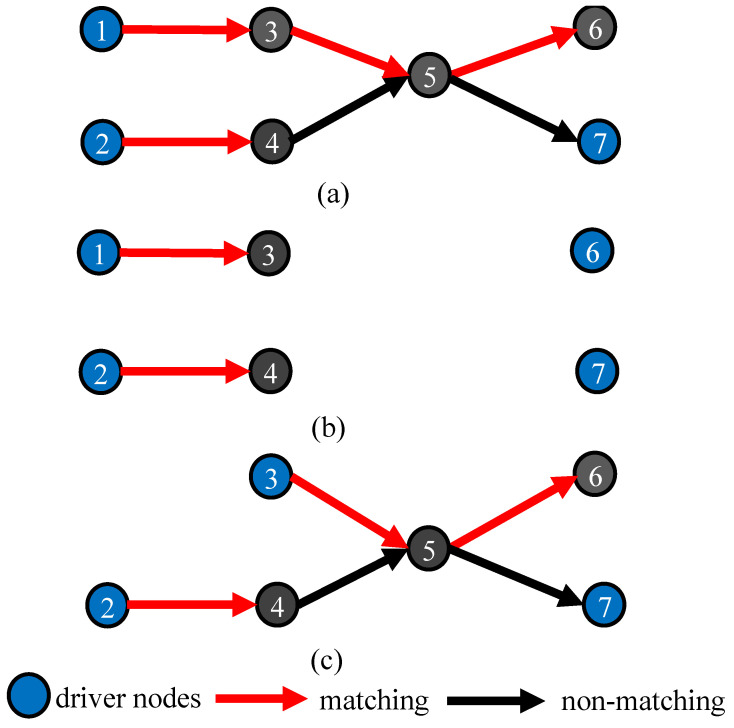
(**b**,**c**) show changes in network driver nodes after attacking critical candidate nodes in (**a**). Attacking non-taboo nodes increases driver nodes (**b**), while attacking taboo nodes shows no change (**c**).

If a first-order critical candidate node ($V_{i}$) is removed, it makes its successor node with an in-degree of 1 become a critical node. As seen in Figure 2a, at least three nodes are required to control the entire network; here, nodes 1, 2, and 7 are used as driver nodes. Nodes 1, 2, and 5 all contain successor nodes with an in-degree of 1, so these three nodes are all first-order critical candidates. After removing node 5, node 6 needs to be added as an additional driver node, while the number of driver nodes in the current network is increased by 1, i.e., a minimum of four nodes (1, 2, 6, and 7) is required to control the entire network, as shown in Figure 2b. Different first-order critical candidate nodes may have different numbers of nodes with an in-degree of 1 among their successor nodes, and removing them produces different numbers of critical nodes.

#### 3.1.2. First-Order Candidate Degree (FCD)

First-order candidate degree is the number of nodes with an in-degree of 1 among the successor nodes of a first-order critical candidate node.

As shown in Figure 2a, the first-order candidate degrees of nodes 1 and 2 are both 1, while that of node 5 is 2, so node 5 should be attacked in priority; during the node-removal attack, it will produce more critical nodes if the first-order critical candidate node with the larger first-order candidate degree is removed.

In addition, the first-order critical candidate nodes in the network may disappear during attacks, or for some networks with a high average degree, there may be no first-order critical candidate nodes in the network. Therefore, it is also necessary to pay attention to other nodes that can disrupt network controllability.

### 3.2. Second-Order Critical Candidate Nodes

When there is no first-order critical candidate node in the network, if there is a node with an in-degree of 2, removing one of its predecessor nodes makes the in-degree of this node decrease to 1 so that another predecessor node of this node becomes a first-order critical candidate node.

#### 3.2.1. Second-Order Critical Candidate Nodes (SCCNs)

If a node in the network has a successor node with an in-degree equal to 2, the node is called a second-order critical candidate node.

Similarly, the greater the number of nodes with in-degree of 2 among the successor nodes of a second-order critical candidate node, the more first-order critical candidate nodes produced after removing this node.

#### 3.2.2. Second-Order Candidate Degree (SCD)

Second-order candidate degree is the number of nodes with an in-degree of 2 among the successor nodes of a second-order critical candidate node.

### 3.3. Critical Candidate Node-Based Attack Strategy

First of all, for some critical nodes, there are nodes with in-degrees of 1 or 2 among their successor nodes, so they are also first- or second-order critical candidate nodes. The size of an MDS does not increase when attacking this type of node, which is called the taboo node, and it should be avoided as much as possible during attacks. In Figure 2a, first-order critical candidate nodes 1 and 2 are also critical (driver) nodes. The remaining network after removing node 1 is shown in Figure 2c; the number of driver nodes does not increase, so both nodes 1 and 2 are taboo nodes. Therefore, before the node-removal attack, all nodes in the original network should be traversed to obtain all the taboo nodes and place them into the taboo list; then, the first- and second-order candidate degrees of nodes in the taboo list should be set to 0 to avoid selecting them as target nodes.

In summary, the priority attack orders in the proposed critical candidate node-based attack model are as follows: (1) the first-order critical candidate nodes of non-taboo nodes in the current network; (2) the second-order critical candidate nodes of non-taboo nodes; (3) if there is no first- or second-order critical candidate node in the current network, the node with the maximum degree (the maximum out-degree for directed networks) is attacked until the first- or second-order critical candidate node appears or until the end of the attack process. The specific program steps are shown in Algorithm 1. We also provide a mathematical proof detailing why attacking non-taboo successor nodes is effective and leads to an increase in the number of driver nodes.
**Algorithm 1** Critical Candidate Node-based Attack**Input:** a network $G_{0}$ with *N* nodes.**Output:** index *t* of the node to be attacked.  1: taboo_list←Array(0)  2: FCD←Array(N)  3: SCD←Array(N)  4: out_degrees←Array(N)  5: **for** i=1 to *N* **do**  6:     **if** $get\_indegree(V_{i})==0$ **then**  7:         taboo_list.insert(i)  8:     **end if**  9: **end for**10: **for** j=1 to *N* **do**11:     **if** *j* in taboo_list **then**12:         FCD[j]←013:         SCD[j]←014:     **else**15:         $FCD\left[j\right]\leftarrow calculate\_FCD\left(V_{j}\right)$16:         $SCD\left[j\right]\leftarrow calculate\_SCD\left(V_{j}\right)$17:     **end if**18:     $out\_degrees\left[j\right]\leftarrow get\_outdegree\left(V_{j}\right)$19: **end for**20: **if** max(FCD)!=0 **then**21:     t←argmax(FCD)22: **else if** max(SCD)!=0 **then**23:     t←argmax(SCD)24: **else**25:     t←argmax(out_degrees)26: **end if**27: **return** *t*

**Theorem** **1.** *After attacking the successor nodes that are non-taboo nodes, the number of driver nodes in the network increases, and the size of the minimum drive node set (MDS) increases; a larger MDS means worse network controllability. Conversely, attacking the successor nodes of taboo nodes, the number of driver nodes remains unchanged.*

**Proof.**  Let $A_{N}$ be the adjacency matrix of a directed graph with *N* nodes. If there exists a node ($v_{i}$) with an out-degree 1 and its successor node is $v_{j}$, then the adjacency matrix is represented as shown in Equation (6), where $D_{N-2}$ is an (N−2)×(N−2) square matrix, *a* and *c* are (N−2)-dimensional column vectors, *b* is an (N−2)-dimensional row vector, 0 denotes an (N−2)-dimensional zero vector, and *d* is either 1 or 0.

(6) AN=  v1v2 ⋯ vjviv1v2 ⋮ vjvi                DN−2  ac                b  0d  0  10

After elementary transformations, Equation (6) is transformed into Equation (7). At this point, the rank of $A_{N}$ can be calculated using Equation (8).

(7) AN=                DN−2  ca                b  d0  0  01

(8) Rank(AN)=1+Rank  DN−2c  bd

Let $N_{D}$ represent the number of driver nodes. The formula for calculating the number of driver nodes is given by Equation (9).

(9) ND=N−Rank(AN)=(N−1)−Rank  DN−2c  bd

Let the adjacency matrix of the network formed after attacking node $v_{j}$ be denoted as $A_{N-1}$, as shown in Equation (10). At this point, the count of driver nodes in the network is ${\left(N-1\right)}_{D}$, with the specific calculation given by Equation (11).

(10) AN−1=  v1v2 ⋯ viv1v2 ⋮ vi              DN−2  c              0  0

(11) (N−1)D=(N−1)−Rank(AN−1)=(N−1)−Rank  DN−2c  00

Due to the validity of Equation (12), we obtain Equation (13).

(12) Rank  DN−2c  bd≥Rank  DN−2c  00

(13) $${\left(N-1\right)}_{D}\geq N_{D}$$

(14) $$x\cdot \left(\begin{array}{cc} D_{N-2} & c \end{array}\right)=\left(\begin{array}{cc} b & d \end{array}\right)$$

When Equation (14) has a solution, Equation (13) takes the ‘=’ sign, meaning that attacking the successor nodes of taboo nodes keeps the number of driver nodes unchanged. When Equation (14) has no solution, Equation (13) takes the ‘>’ sign, meaning that attacking the successor nodes of non-taboo nodes increases the number of driver nodes. Therefore, the theorem is proven. □

## 4. Numerical Experimental Studies

To verify the effectiveness of the proposed attack model, it is compared with node centrality-based attack models, which namely degree-, betweenness-, *closeness*, and pagerank-based attack strategies. It is also compared with hierarchical attack (HA) [20], which contributes state-of-the-art disruptive performance to network controllability. In the process of HA, all nodes are divided into four categories, namely critical nodes, subcritical nodes, normal nodes, and redundant nodes. The nodes in the first category are attacked in priority; if the first category is empty, then the nodes in the second category are attacked, and so on. Since HA requires calculating the impact of each node on the network after removal, it is time-consuming and difficult to apply to large-scale networks.

Six representative synthetic network models are used for attack simulation, including Erdös-Rényi (ER) random graph [26], Newman–Watts small-world (SW) [27], generic scale-free (SF) [28], *q*-snapback (QS) [29], random triangle (RT), and random rectangle (RR) [30]. Two real-world networks are also tested, namely *Roget* and *inf-euroroad* [31]. The controllability robustness (*R*) calculated by Equation (5) is used to evaluate the performance of all compared attack models. A higher value of *R* means worse overall controllability robustness under an attack model; it also indicates that the attack model is more destructive.

To eliminate the effect of randomness, each experimental case is run independently and repeated 30 times. The averaged results are reported, and all experiments are performed on a PC with Intel(R) Core i7-6700 CPU, which has 8 GB memory running a Windows10 Operating System.

### 4.1. Attack Simulation on Synthetic Networks of Different Sizes

In general, large-scale networks are relatively more complex and sufficient in terms of structure, and the network size usually affects the network controllability robustness, i.e., more driver nodes are usually required for a larger network when the average degree (〈k〉) is the same. Also, for node-removal attacks, computation on large-scale networks is more time-consuming.

In this experiment, three different sizes of networks are tested, namely 500, 1000, and 1500. There are a total of 3×6=18 instances, namely three network sizes and six synthetic network models; each instance keeps the same average degree of 〈k〉=5.

**Figure 3:**
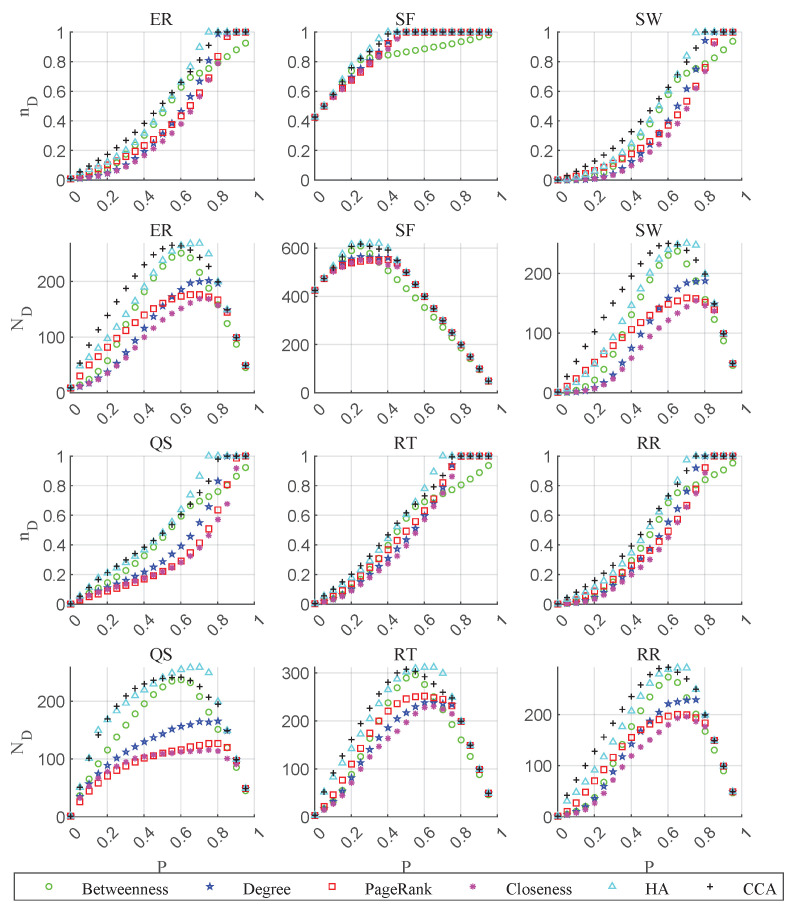
Results of attack simulation on networks with N=1000 and 〈k〉=5, $n_{D}$ is calculated by Equation (1) and $N_{D}$ is calculated by Equation (2), *P* is the proportion of removed nodes.

Figure 3 illustrates the change in controllability ($n_{D}$) calculated by Equation (1) and the minimum number of drive nodes ($N_{D}$) calculated by Equation (2) during node-removal attacks. $n_{D}$ depends on the minimum number of drive nodes ($N_{D}$) and the network size (*N*); *N* decreases during attacks. Therefore, $n_{D}$ is equal to 1 at some point, which means that all nodes of the current network are driver nodes, i.e., all nodes are isolated. The rising curve of $n_{D}$ proves that the attacks are effective. For $N_{D}$, the curve starts to decrease with network size when all nodes are isolated (driver nodes).

Table 1 lists the overall controllability robustness (*R*) calculated by Equation (5) for all cases. For all 18 comparison cases, CCA obtains a total of 11 best performances, while HA obtains 7 and the others obtain 0. Averaging the results across the six types of networks, CCA achieves the best performance at all three network sizes, and HA performs worse than CCA but better than the other methods.

Table 2 shows the time consumption of all methods on three different network sizes. It is clear that degree-, pagerank-, and closeness-based attacks cost very little time, while betweenness-based attacks and HA cost relatively more time and CCA takes less time than both HA and betweenness-based attacks. According to Table 1, for the other methods, performance and time consumption are often proportional, but the proposed CCA achieves the best performance while greatly reducing time complexity.

### 4.2. Attack Simulation on Synthetic Networks of Different Average Degrees

It is pointed out that sparse and heterogeneous networks are harder to control and less robust in network controllability, while dense and homogeneous networks are easy to control and have better robustness in network controllability [4]. So a network with a large average degree 〈k〉 always has good controllability robustness, and it is correspondingly difficult to disrupt the network controllability by malicious attacks.

In this experiment, all networks keep the same size N=1000, while consisting of 4 groups, in each group, the average degrees are 3, 5, 7, and 10 respectively. So, there are a total of 4×6=24 instances, namely 4 average degrees and 6 synthetic network models, and each instance keeps the same network size N=1000.

As shown in Table 3, for any type of attack, the controllability robustness *R* decreases continuously as the average degree of the networks grows, which also indicates that the network becomes more resistant to attacks and more robust. Nevertheless, the CCA model is highly adaptive and can still disrupt the network controllability for different average degrees efficiently, and the larger the average degree the more obvious the effectiveness of CCA. Specifically, for the overall ranking, the attack effectiveness of CCA is slightly inferior to that of HA for an average degree of 〈k〉=3, while better in all other cases.

### 4.3. Attack Simulation on Real-World Networks

In this experiment, two real-world networks are used to test the attack models, namely *Roget* (1022 nodes and 5075 edges) and *inf-euroroad* (1175 nodes and 1417 edges).

As shown in Table 4, CCA achieves the best performance on both real-world networks when considering both effectiveness and time complexity. Specifically, for the attack model HA, which has similar effectiveness with CCA but takes about 453 and 447 times longer; for the other models, the time consumptions are similar, but the attack effectiveness is far less than that of CCA.

### 4.4. Visualization of Target Node Selection during Attack Simulation

In this experiment, two ER networks with average degrees of 〈k〉=5 and 〈k〉=10 are attacked by CCA; both networks consist of N=500 nodes. The selected target node in each step during attacks is recorded, and whether it is a first-order critical candidate node, second-order critical candidate node, or a node with a maximum degree is visualized.

**Figure 4:**
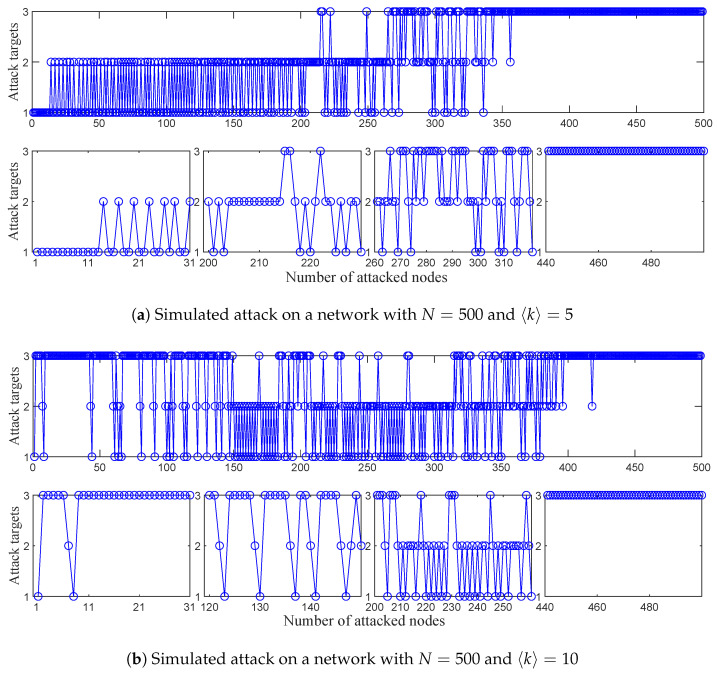
Visualization of the CCA process.

The complete attack process is visualized in Figure 4, where 1, 2, and 3 in the vertical coordinates represent the attack on the first-order critical candidate nodes, second-order critical candidate nodes, and nodes with maximum degree, respectively. As shown in Figure 4a, there are initially 10 first-order critical candidate nodes. After removing all first-order critical candidate nodes, the second-order critical candidate nodes start to be attacked, and after that, the first-order critical candidate nodes can be produced, so first- and second-order critical candidate nodes are alternately attacked until there are no second-order critical candidate nodes. Finally, as before, the first- and second-order critical candidate nodes are also produced when the node with the maximum degree is attacked, so the first- and second-order critical candidate nodes, was well as nodes with the maximum degree are alternately attacked until all nodes are isolated.

As shown in Figure 4b, for a network with a high average degree, there are rarely critical candidate nodes, so a degree-based attack is used preferentially to produce a large number of first- or second-order critical candidate nodes until all nodes in the network are isolated.

## 5. Conclusions

Assessing and disrupting network controllability is crucial for understanding and enhancing network resilience against malicious attacks. In this paper, a novel attack model based on critical candidate nodes is proposed. By categorizing nodes into first-order and second-order critical candidate nodes according to the in-degrees of their successor nodes, this approach identifies and targets nodes pivotal for maintaining network controllability. This method not only improves the efficiency of node-removal attacks by minimizing computation time but also ensures maximal disruption compared to traditional methods.

Extensive simulations on both synthetic and real-world networks demonstrate the superiority of the proposed model in effectively reducing network controllability. The findings highlight the significant impact of critical candidate nodes in maintaining network robustness, providing a new perspective for understanding and enhancing network resilience against malicious attacks.

However, this study also has certain limitations. The current model primarily focuses on directed networks, and its applicability to undirected or weighted networks requires further investigation. Future work could explore adaptive attack strategies that account for dynamic network changes and the development of defense mechanisms to counteract such targeted attacks.

## Figures and Tables

**Table 1:** Results of attack simulation on networks of different sizes with 〈k〉=5.

Network	Attack	N=500		N=1000		N=1500	
		**R**	**Rank**	**R**	**Rank**	**R**	**Rank**
ER	Betweenness	0.4565	3	0.4535	3	0.4544	3
	Degree	0.4277	4	0.4266	4	0.4264	4
	Pagerank	0.4269	5	0.423	5	0.425	5
	Closeness	0.3866	6	0.3831	6	0.3832	6
	HA	0.5265	2	0.5257	2	0.5244	2
	CCA	0.5406	1	0.5408	1	0.5412	1
SF	Betweenness	0.8093	6	0.8267	6	0.8379	6
	Degree	0.8526	3	0.8735	3	0.8843	3
	Pagerank	0.8434	4	0.8669	4	0.8782	4
	Closeness	0.8395	5	0.8624	5	0.8748	5
	HA	0.8771	1	0.8956	1	0.9047	1
	CCA	0.8705	2	0.8902	2	0.8996	2
SW	Betweenness	0.4159	3	0.417	3	0.4148	3
	Degree	0.3806	5	0.3851	4	0.3858	4
	Pagerank	0.3812	4	0.3807	5	0.3815	5
	Closeness	0.3322	6	0.3359	6	0.3362	6
	HA	0.4742	2	0.4804	2	0.4753	2
	CCA	0.5072	1	0.5105	1	0.5121	1
QS	Betweenness	0.4627	2	0.4616	3	0.4599	3
	Degree	0.4141	3	0.4123	4	0.4137	4
	Pagerank	0.3394	4	0.3453	5	0.3471	5
	Closeness	0.332	5	0.3301	6	0.3281	6
	HA	0.332	5	0.547	1	0.5483	1
	CCA	0.5289	1	0.5295	2	0.5306	2
RT	Betweenness	0.4946	3	0.4971	4	0.4976	5
	Degree	0.4902	4	0.497	5	0.4977	4
	Pagerank	0.52	2	0.5234	3	0.5232	3
	Closeness	0.4711	5	0.476	6	0.4752	6
	HA	0.4711	5	0.588	1	0.5865	1
	CCA	0.5808	1	0.5829	2	0.5822	2
RR	Betweenness	0.4662	2	0.4666	3	0.4646	3
	Degree	0.4583	3	0.4601	4	0.4597	4
	Pagerank	0.4464	4	0.4478	5	0.4505	5
	Closeness	0.4115	5	0.4135	6	0.4144	6
	HA	0.4115	5	0.5425	2	0.5433	2
	CCA	0.5598	1	0.5596	1	0.5618	1
Avg.	Betweenness	0.518	2	0.52	3	0.522	3
	Degree	0.504	4	0.509	4	0.511	4
	Pagerank	0.493	5	0.498	5	0.501	5
	Closeness	0.462	6	0.467	6	0.469	6
	HA	0.515	3	0.597	2	0.597	2
	CCA	0.598	1	0.602	1	0.605	1

**Table 2:** Run time of attack simulations on networks of different sizes with 〈k〉=5.

Attack	N=500	N=1000	N=1500
Betweenness	4.23 (s)	33.72 (s)	114.78 (s)
Degree	0.15 (s)	1.21 (s)	4.06 (s)
Pagerank	0.49 (s)	2.37 (s)	6.32 (s)
Closeness	0.62 (s)	3.94 (s)	10.69 (s)
HA	50.49 (s)	720.22 (s)	7389.36 (s)
CCA	1.62 (s)	5.59 (s)	14.30 (s)

**Table 3:** Results of attack simulation on networks of different average degree 〈k〉.

Network	Attack	〈k〉=3		〈k〉=5		〈k〉=7		〈k〉=10	
		**R**	**Rank**	**R**	**Rank**	**R**	**Rank**	R	**Rank**
ER	Betweenness	0.5889	3	0.4535	3	0.3723	3	0.2944	3
	Degree	0.5808	4	0.4266	4	0.3323	5	0.2584	5
	Pagerank	0.5588	5	0.423	5	0.349	4	0.2807	4
	Closeness	0.5377	6	0.3831	6	0.2955	6	0.2273	6
	HA	0.6798	1	0.5257	2	0.4276	2	0.3322	2
	CCA	0.6659	2	0.5408	1	0.4505	1	0.3557	1
SF	Betweenness	0.8751	6	0.8267	6	0.7813	6	0.7216	6
	Degree	0.9256	3	0.8735	3	0.8236	3	0.755	3
	Pagerank	0.9218	4	0.8669	4	0.8131	4	0.7429	4
	Closeness	0.9193	5	0.8624	5	0.8095	5	0.736	5
	HA	0.9389	1	0.8956	1	0.8526	1	0.7941	1
	CCA	0.9355	2	0.8902	2	0.8454	2	0.7837	2
SW	Betweenness	0.5096	3	0.417	3	0.3513	3	0.2863	3
	Degree	0.4873	4	0.3851	4	0.316	5	0.2485	5
	Pagerank	0.4398	5	0.3807	5	0.3276	4	0.2686	4
	Closeness	0.4159	6	0.3359	6	0.276	6	0.2152	6
	HA	0.5953	2	0.4804	2	0.3935	2	0.3216	2
	CCA	0.6145	1	0.5105	1	0.4287	1	0.3427	1
QS	Betweenness	0.5416	3	0.4616	3	0.4074	3	0.3458	3
	Degree	0.5069	4	0.4123	4	0.3527	4	0.2888	4
	Pagerank	0.4195	6	0.3453	5	0.2809	5	0.2144	6
	Closeness	0.4213	5	0.3301	6	0.271	6	0.2192	5
	HA	0.6316	1	0.547	1	0.4793	1	0.4038	1
	CCA	0.6183	2	0.5295	2	0.4571	2	0.3772	2
RT	Betweenness	0.6266	6	0.4971	4	0.4137	4	0.3307	4
	Degree	0.648	4	0.497	5	0.3987	5	0.3061	5
	Pagerank	0.6791	3	0.5234	3	0.4255	3	0.3374	3
	Closeness	0.6382	5	0.476	6	0.376	6	0.2841	6
	HA	0.7197	1	0.588	1	0.4874	2	0.3799	2
	CCA	0.6972	2	0.5829	2	0.4998	1	0.4067	1
RR	Betweenness	0.583	3	0.4666	3	0.388	3	0.3128	3
	Degree	0.5787	4	0.4601	4	0.3745	5	0.295	5
	Pagerank	0.5546	5	0.4478	5	0.3761	4	0.3039	4
	Closeness	0.5339	6	0.4135	6	0.3331	6	0.2587	6
	HA	0.6577	1	0.5425	2	0.4525	2	0.3589	2
	CCA	0.6542	2	0.5596	1	0.4795	1	0.3858	1
Avg.	Betweenness	0.621	3	0.52	3	0.452	3	0.382	3
	Degree	0.621	3	0.509	4	0.433	4	0.359	4
	Pagerank	0.596	5	0.498	5	0.429	5	0.358	5
	Closeness	0.578	6	0.467	6	0.394	6	0.323	6
	HA	0.704	1	0.597	2	0.515	2	0.432	2
	CCA	0.698	2	0.602	1	0.527	1	0.442	1

**Table 4:** Results of attack simulation on real-world networks.

Roget (N=1022, 〈k〉=4.97)			
**Attack**	**R**	**Rank**	**Time**
Betweenness	0.4998	6	40.65 (s)
Degree	0.565	4	2.12(s)
Pagerank	0.6172	3	4.83 (s)
Closeness	0.515	5	3.35 (s)
HA	0.6435	2	2737.55 (s)
CCA	0.6465	1	6.04 (s)
inf-euroroad (N=1175, 〈k〉=1.2)			
Attack	R	Rank	Time
Betweenness	0.7181	4	41.89 (s)
Degree	0.7511	3	2.12 (s)
Pagerank	0.6498	6	3.47 (s)
Closeness	0.7005	5	4.89 (s)
HA	0.8104	1	2745.19 (s)
CCA	0.7945	2	6.14 (s)

## Data Availability

The original contributions presented in the study are included in the article, further inquiries can be directed to the corresponding author.
